# Role of the P2 × 7 receptor in neurodegenerative diseases and its pharmacological properties

**DOI:** 10.1186/s13578-023-01161-w

**Published:** 2023-12-13

**Authors:** Ziyan Hu, Yifan Luo, Jinxi Zhu, Danling Jiang, Zhenzhong Luo, Lidong Wu, Jin Li, Shengliang Peng, Jialing Hu

**Affiliations:** 1 Department of Emergency medicine, The Second Affiliated Hospital of Nanchang University, Nanchang, 330006 China; 2 Department of the second Clinical Medical College, Nanchang University, Nanchang, 330006 China; 3 Department of Ultrasound Medicine, The Second Affiliated Hospital of Nanchang University, Nanchang, 330006 China; 4 Department of Anesthesiology, The Second Affiliated Hospital of Nanchang University, Nanchang, 330006 China

**Keywords:** P2 × 7R, ATP, Neurodegenerative Disease, Antagonists, Pharmacology

## Abstract

Neurodegenerative diseases seriously affect patients’ physical and mental health, reduce their quality of life, and impose a heavy burden on society. However, their treatment remains challenging. Therefore, exploring factors potentially related to the pathogenesis of neurodegenerative diseases and improving their diagnosis and treatment are urgently needed. Recent studies have shown that P2 × 7R plays a crucial role in regulating neurodegenerative diseases caused by neuroinflammation. P2 × 7R is an adenosine 5′-triphosphate ligand-gated cation channel receptor present in most tissues of the human body. An increase in P2 × 7R levels can affect the progression of neurodegenerative diseases, and the inhibition of P2 × 7R can alleviate neurodegenerative diseases. In this review, we comprehensively describe the biological characteristics (structure, distribution, and function) of this gene, focusing on its potential association with neurodegenerative diseases, and we discuss the pharmacological effects of drugs (P2 × 7R inhibitors) used to treat neurodegenerative diseases.

## Background

When the neurons of the central and peripheral nervous systems are no longer structurally or functionally normal, a group of diseases directly caused by protein abnormalities develop. The deposition of amyloid proteins and network degradation are hallmarks of most neurodegenerative diseases (NDs). NDs have heterogeneous clinical and pathological characteristics, but some traits overlap. Based on clinical manifestations, NDs can be broadly categorized into different types, with extrapyramidal or pyramidal movement disorders and cognitive or behavioral impairments being the most prevalent types [1–5]. Representative NDs include Alzheimer’s disease (AD), Parkinson’s disease (PD), Huntington’s disease (HD), and amyotrophic lateral sclerosis (ALS) [6]. Abnormal aggregation of a diverse range of proteins leads to NDs, for which the standard diagnosis is currently neuropathological evaluation at autopsy [2, 7]. Insights into treatments, such as the modulation of autophagy, stem cell-derived exosomes, and gene therapy, some of which are reviewed in the following sections, have been reported [7–9]. Although a proven therapeutic schedule and an applicable and systematic method have not yet been developed, the important role of P2 × 7R in mediating NDs and the significance of P2 × 7R as a potential treatment target for NDs are discussed in this review.

P2 × 7R is an adenosine 5′-triphosphate (ATP)-binding ligand-gated ion channel receptor and a member of the P2X family that is activated by high ATP concentrations [10, 11]. P2 × 7R is found in the neurons and glial cells of the brain (astrocytes, oligodendrocytes, and microglia) [12]. P2 × 7R has been shown to be involved in different NDs. For example, depression due to chronic unpredictable mild stress (CUMS) was found to activate neuroinflammation via the P2 × 7R pathway, increasing susceptibility to PD. Moreover, P2 × 7R expression levels have been found to be associated with amyloid beta (Aβ) protein plaques, which lead to the development of AD [13, 14]. Additionally, P2 × 7R mediates the development of NDs by promoting the inflammatory response [15, 16].

In this review, we provide a comprehensive overview of the potential to target P2 × 7R for the treatment of NDs, and we discuss the pharmacological properties of drugs used to treat NDs. The structure and functions of P2 × 7R are also discussed. Finally, we discuss the role of P2 × 7R in NDs and the nervous system, its mechanism of action, and the application of receptor antagonists for the treatment of NDs.

## Structure of P2 × 7R

P2 × 7R, a member of the ligand-gated cationic channel P2X receptor family that evolved from P2 purinoceptors, is an ATP-gated nonselective cationic channel with three subunits that allow Ca2^+^, K^+^, and Na^+^ to pass through the plasma membrane and participate in neuroinflammation [17–19]. It is pharmacologically characterized by its low affinity toward ATP (EC50 > 100 μM) [20]. In mitochondria, oxidative phosphorylation produces ATP. ATP is released into the extracellular space in response to both normal and pathological events [21]. P2 × 7R has unusual permeability characteristics and is expressed in a wide range of cell types, primarily in the immune system, where it plays key roles in cytokine release, oxygen radical production, and T-lymphocyte differentiation and proliferation [22]. It is a vital receptor in neuroinflammation. P2 × 7R was the first P2R family member to be discovered, and it is involved in the immune response [8]. It is expressed in the hematopoietic immune and intrinsic cells of the nervous system, including macrophages, microglia, neurons, astrocytes, and oligodendrocytes [23].

The human *P2 × 7* gene is found on the long arm of chromosome 12 at 12q24.31. It encodes a receptor of 595 amino acids comprising three subunits, with no established heterotrimeric structures with other P2X isoforms, such as P2XI, P2 × 2, P2 × 3, P2 × 6. P2RX7 is centromeric, and close to P2RX4 [24].

**Fig. 1:**
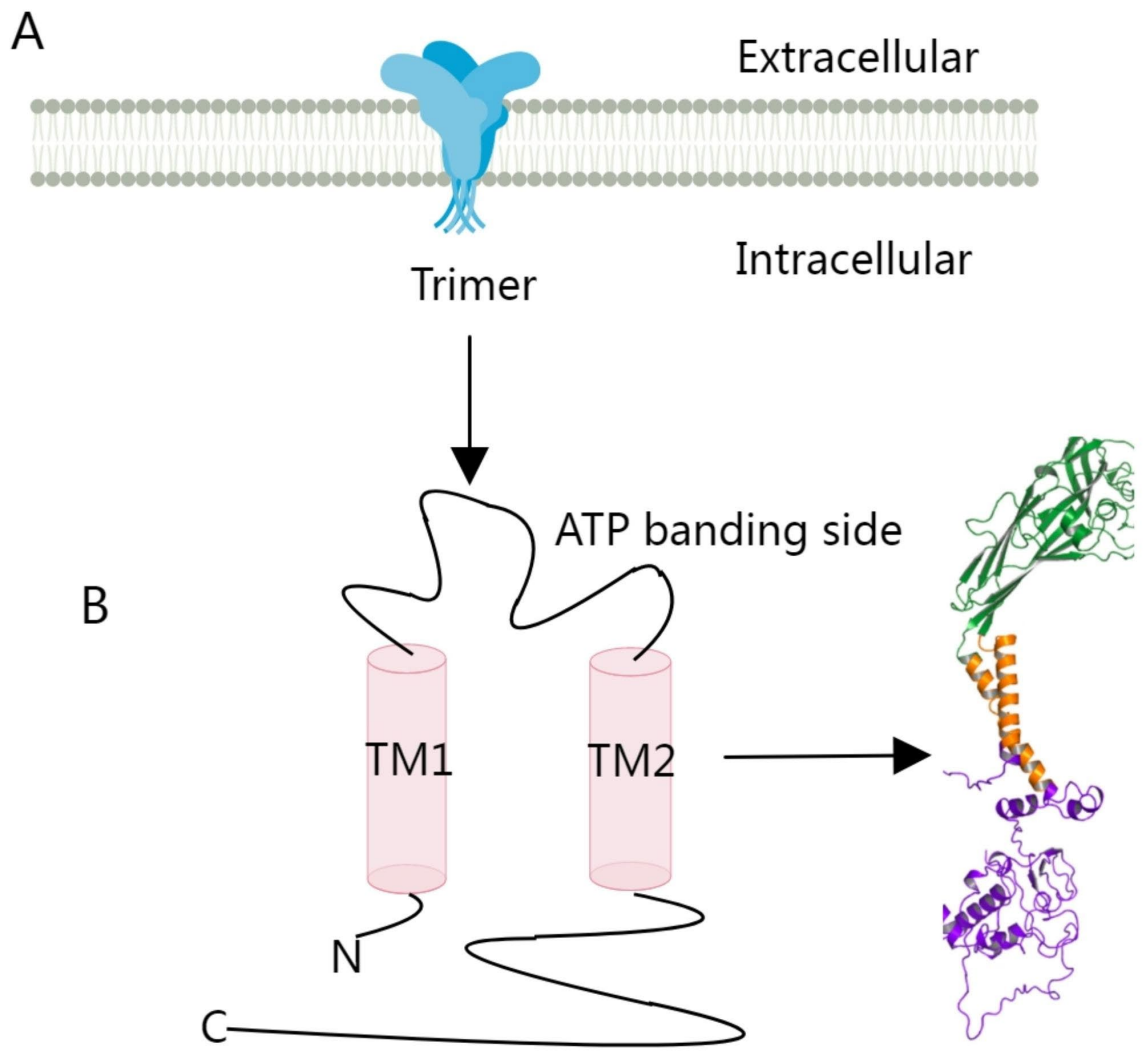
Schematic diagram of the basic structure of P2 × 7R. **A** and **B** show the extracellular region and two transmembrane helices (TM1 and TM2) of P2 × 7R. The receptor is composed of two ATP-binding sites, two alpha-helical transmembrane domains, an intracellular N-terminus, an extended C-terminal tail, and a large extracellular loop. Figure 1 has been modified based on Zhang WJ et al. [31]

Therefore, the unique structure of P2 × 7R is crucial for its function. The functional receptor is composed of a trimeric subunit and its structure is composed of an ionic pore with six transmembrane (TM) domains for each subunit, a longer intracellular domain, and three ATP-binding sites in a chalice-like extracellular domain [25]. The three-dimensional structure of the P2 × 7R subunit is dolphin-shaped, with the extracellular region serving as the body (including the head and fins) and two transmembrane helices (TM1 and TM2) serving as the tail [26]. The 595-amino-acid P2 × 7R subunit or monomer encoded by *P2RX7* combines to create a functional receptor [18, 27]. Human P2 × 7R is composed of two ATP-binding sites, two alpha-helical transmembrane domains, an intracellular N-terminus and an extended C-terminal tail, and a large extracellular loop (Fig. 1) [28, 29]. Recent studies have investigated the relationship between the N-terminus of P2 × 7R and calcium influx using patch-clamp fluorescence assays. It was discovered that the total calcium current was controlled by the N-terminal sequence and that various sites at the N-terminus had different permeabilities to calcium. The longest transmembrane domain in the P2X family is the C-terminus, which contains 293 amino acids. It can activate downstream signaling pathways and facilitate attachment to other proteins [27]. The intracellular C-terminus of P2 × 7R contains additional amino acids. High quantities of ATP create large pores that release inflammatory cytokines and cause apoptotic cell death, whereas low doses open cation channels, occasionally resulting in cell proliferation [30].

## Electrophysiology and related signaling pathways of P2 × 7R

As a subtype of the P2X purinoceptor family, the ligand-gated nonspecific cation channel P2 × 7R is activated by ATP and its derivatives [32]. Notably, the activation efficiencies of different types of P2 × 7R activators vary. For example, 3′-O-(4-benzoyl)benzoyl ATP (BzATP) has a 10–30 times higher activation efficiency than ATP [33, 34].

Unlike other subtypes of the family that are stimulated by micromolar concentrations of ATP, P2 × 7R is activated by high concentrations of ATP (in the millimolar range) that are usually observed under conditions of stress and tissue damage, suggesting a major role of P2 × 7R in inflammation [21, 35].

Upon activation, P2 × 7R undergoes molecular conformational changes, leading to the opening of ion channels on the cell membrane for small cations, including Ca^2+^, Mg^2+^, Na^+^, and K^+^ [17, 36]. Under longer ATP stimulation times (within seconds to minutes), the ion channel expands, allowing molecules smaller than 900 Da to pass through [19]. Moreover, persistent activation of P2 × 7R leads to the formation of pannexin pores, allowing further extracellular release of ATP, which may be related to cytotoxicity and cell death [19, 37].

**Fig. 2:**
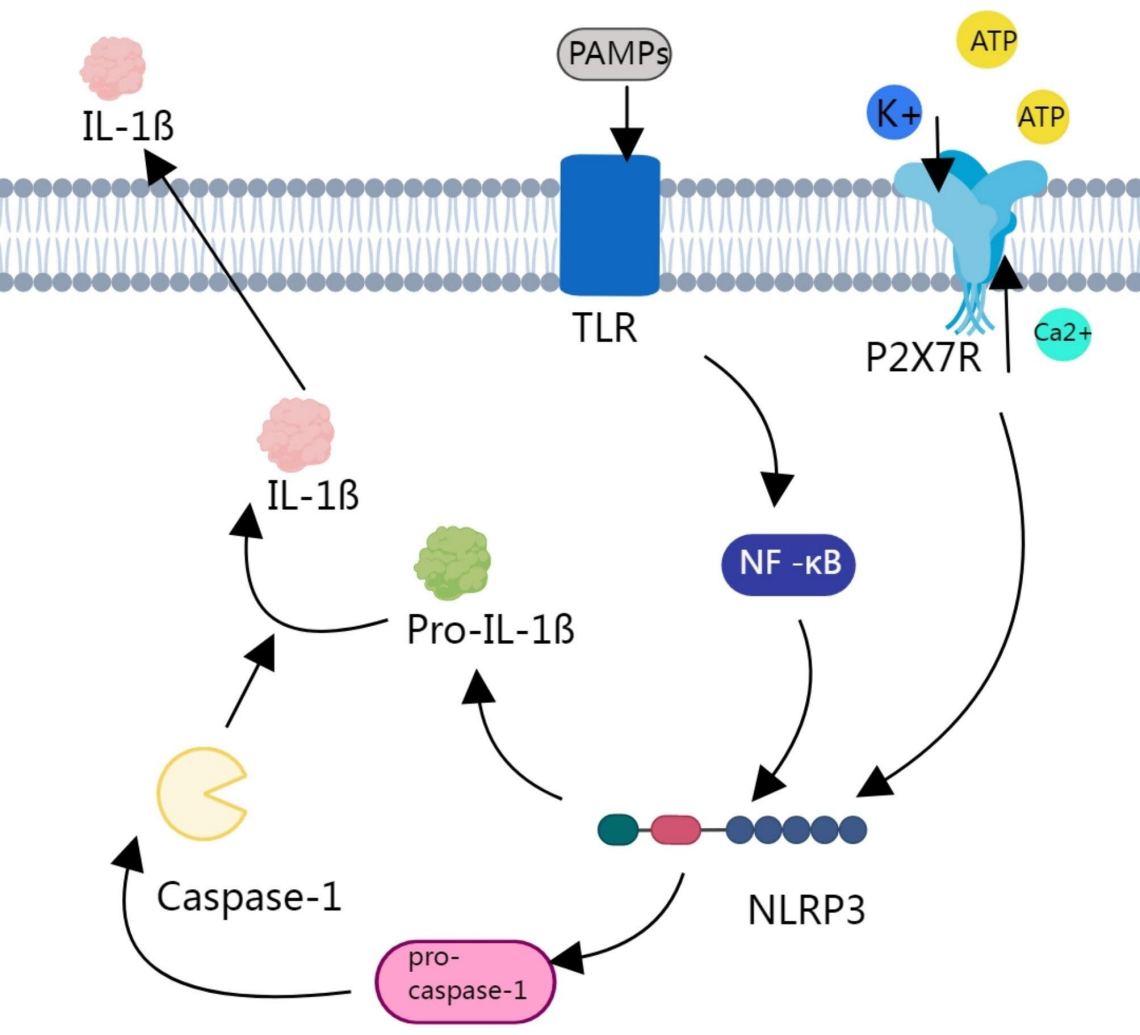
Schematic diagram of the P2 × 7R/TLR pathway in the mediation of inflammation. High concentrations of ATP activate P2 × 7R and open the ion channels to mediate Ca^2+^ influx and K^+^ efflux, which stimulate the assembly of the NLRP3 inflammasome. Both TLR and P2 × 7R activate the NLRP3 inflammasome, with TLR performing this function via the NF-κB pathway. The NLRP3 inflammasome converts pro-IL-1ß to active IL-1ß. The NLRP3 inflammation also plays a role in the conversion of pro-caspase-1 to caspase-1. The two pathways work together to secrete IL-1ß

The activation of P2 × 7R plays a key role in neuroinflammatory processes. Its activation is related to several signaling pathways, including the nuclear factor kappa beta (NF-κB), NLRP3, and mitogen-activated protein kinase (MAPK) pathways (Fig. 2), which are involved in the modulation of astroglial and microglial cell phenotypes and functions and, ultimately, affect the occurrence and development of numerous central nervous system (CNS) diseases [38, 39].

At the initial stage of the inflammatory response, damage-associated and pathogen-associated molecular patterns activate pattern recognition receptors, such as Toll-like receptors (TLRs), which are expressed on immune cells. TLR activation mediates the NF-κB pathway, which acts as the first signal promoting the transcription of several genes that encode for inflammatory mediators, such as inflammasome components and pro-IL1β [40].

P2 × 7R stimulation represents the second signal and is based on the P2 × 7R/NLRP3 interaction. As an important member of the NOD-like receptor family, NLRP3 plays a mediating role in P2 × 7R signaling and the neuroinflammatory changes induced by P2 × 7R. After activation by high levels of ATP released from neurons or astrocytes, P2 × 7R elicits K^+^ efflux, which, in turn, leads to NLRP3 inflammasome activation. The NLRP3 inflammasome mediates the activation of caspase-1, a member of the inflammatory caspase family, which then converts nonactive pro-caspase-1 to cleaved caspase-1 (P20), which further converts inactive pro-IL-1b and pro-IL-18 to active IL-1b and IL-18, respectively [41]. A study focusing on the development of tolerance to morphine-induced antinociception indicated that both TLR4- and P2 × 7R-dependent pathways are required for NLRP3 inflammasome activation, further supporting the “two-step” theory of the NLRP3 inflammasome [42].

The important role of the NF-κB pathway in the relationship between the TLR4- and P2 × 7R-dependent pathways has also been confirmed by an increasing number of reports linking pro-inflammatory activity of P2 × 7 to NF-κB nuclear translocation [43, 44]. However, the mechanism underlying inflammatory factor generation during neuroinflammation requires further investigation. Subtle, but noteworthy, differences may exist in the occurrence and development of different NDs. Studies have shown that P2 × 7R/SFK signaling may be involved in the pathophysiology of cortical-spreading-depression-associated migraine [45].

MAPKs are a group of serine-threonine protein kinases that can be activated by different extracellular stimuli. Six MAPK subfamilies have been identified and cloned into mammalian cells: extracellular signal-regulated kinase (ERK)1/2, Jun N-terminal kinase (JNK), ERK3, p38s, ERK5, and ERK7/8. MAPKs are closely related to the occurrence and development of CNS diseases, such as AD and PD. Mononuclear cells of patients with newly diagnosed PD display hyperexpression of the P2 × 7R/NLRP3 inflammasome platform, which seems to modulate cellular synuclein content. Impaired JNK phosphorylation may be an intracellular signal that mediates this effect [46]. Another study showed that P2 × 7R-dependent expression of ATP-induced pro-inflammatory tumor necrosis factor-α (TNF-α) is regulated in microglial cells by ERK and c- JNK [47].

Previous studies have also suggested that P2 × 7R may act as a central mediator between pathways involved in the development of neuroinflammation, while the P2 × 7R antagonist, Brilliant Blue G (BBG), inhibits the activation of MAPKs by inhibiting the phosphorylation of p38 MAPK and c-JNKs and the nuclear translocation of the transcription factor p65, leading to the inactivation of NF-κB [48]. In summary, P2 × 7R plays a pivotal role in signaling pathways related to neuroinflammatory responses. Therefore, P2 × 7R-based targeted therapies may play an important role in the treatment of neurological diseases.

## P2 × 7R antagonists and their pharmacological properties

P2 × 7R-induced neuroinflammation influences the development of a wide range of CNS disorders, such as spinal cord injury; intracerebral hemorrhage; major depression; bipolar disorder; and NDs, including AD, PD, ALS, and HD [41–44].

Therefore, P2 × 7R is considered an appropriate target for the treatment of several neurodegenerative and neurological diseases, as well as mood disorders. Further exploration of potent P2 × 7R antagonists may facilitate the development of drugs for the treatment of P2 × 7R-associated diseases.

Based on the history of P2 × 7R antagonist development, we classified the antagonists into two types according to their chemical composition: ATP-derivative antagonists and non-ATP-based compounds. ATP-derivative antagonists include 2′,3′-O-trinitrophenyl-ATP and periodate-oxidized ATP. Non-ATP-based compounds can be further subdivided, depending on their interaction with the receptor, into orthostatic antagonists that competitively bind to the ATP-binding pocket and allosteric antagonists that bind to locations other than the ATP-binding site to reduce ATP-binding affinity [49].

BBG is currently the most widely used P2 × 7R antagonist, as it is inexpensive, readily available, and widely used in mechanistic research on P2 × 7R in nervous system diseases. A study using human *SOD1*^G93A^ transgenic mice investigated whether BBG could alter disease progression in this ALS mouse model. BBG treatment was found to reduce weight loss and prolong survival in female *SOD1*^G93A^ mice [50]. However, BBG has poor selectivity toward P2 × 7R and it is a large, charged molecule with unclear permeability across the blood-brain barrier (BBB) [51, 52]. Recent studies have provided additional insights into the important role of P2 × 7R in neuroinflammation and, subsequently, in NDs, and researchers are currently searching for more potent and selective P2 × 7R antagonists that target the CNS with BBB permeability. Several new P2 × 7R antagonists have been developed that exploit various scaffolds and molecular systems.

For example, JNJ-54,175,446 and JNJ-55,308,942 are novel, selective, and brain-penetrant P2 × 7R antagonists that have recently been reported. A double-blind, placebo-controlled translational study assessed the safety and tolerability of multiple doses of JNJ-54,175,446 and explored its effects on PD using dexamphetamine challenge. All participants showed high tolerance to JNJ-54,175,446 at all tested doses and it suppressed the ex vivo lipopolysaccharide (LPS)-induced release of cytokines [53]. Evaluation based on an animal model of neuroinflammation and pleasure deficit showed that JNJ-55,308,942 attenuated IL-1β release in human blood, mouse blood, and mouse microglia isolated from pup brains, as expected. This compound has been shown to effectively prevent Bz-ATP-induced cell death in a concentration-dependent manner [54].

A804598, which is based on (R)-α-methyl benzylamine, is another promising P2 × 7R antagonist with excellent potency and outstanding pharmacokinetic properties. Recently, by administering different doses of A804598 to C57BL/6J mice exposed to chronic ethanol and a high-fat diet, researchers found that A804598 treatment reversed the changes in microglia and astrocytes; reduced or abolished increases in the mRNA levels of several inflammatory markers, including IL-1β, iNOS, CXCR2, and components of inflammatory signaling pathways, such as TLR2, CASP1, NF-κB1, and CREB1; and controlled the protein levels of pro-IL-1β and NF-κB1 [55]. Chronic in vivo treatment with A-804,598 in *SOD1*^G93A^ mice was shown to inhibit the upregulation of SQSTM1/p62 in the lumbar medulla by inhibiting P2 × 7R, confirming that P2 × 7 is an in vivo regulator of autophagy in the pathogenesis of ALS [56].

**Fig. 3:**
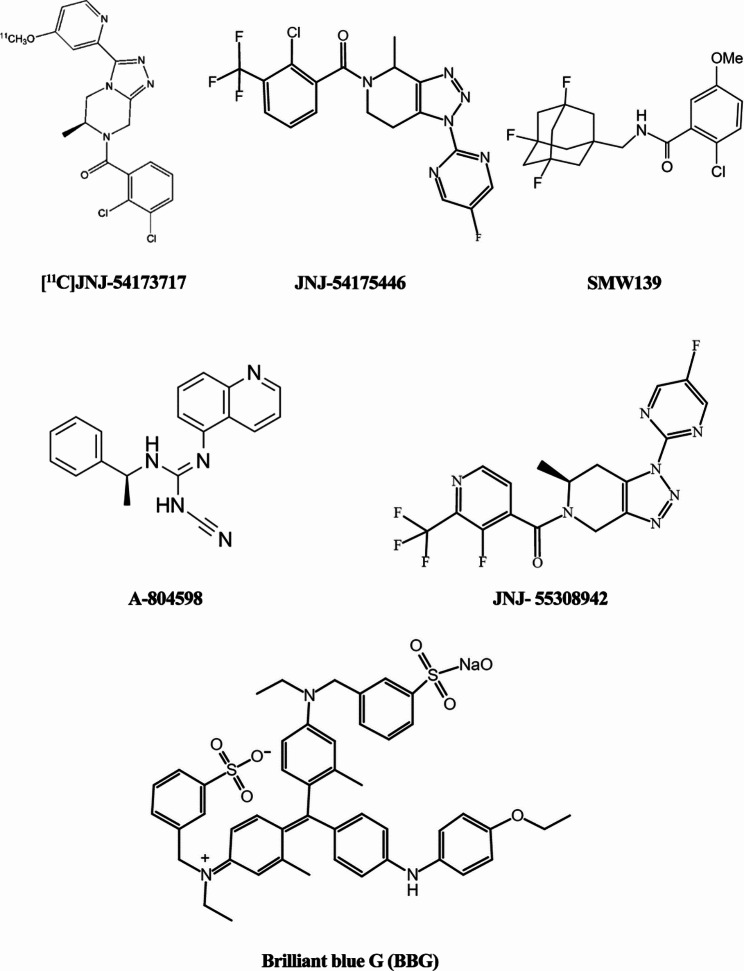
Molecular structures of commonly used P2 × 7R antagonists

With the increasing amount of research on P2 × 7R antagonists, many new ligands have been evaluated in different models of CNS disorders for which P2 × 7R has been implicated (Fig. 3; Table 1). However, only a few ligands have successfully entered clinical trials and further studies on the structure of P2 × 7R are required. The new generation of P2 × 7R antagonists requires not only appropriate properties targeting the CNS, that is, adequate lipophilicity, water solubility, and a long half-life in brain tissue, but also high specificity for P2 × 7R, to further clarify the mechanism of action and the exact function of P2 × 7R in the CNS. In addition to specificity, minimizing the differences in agonist potency across mammalian species is also a factor to consider in studies of P2 × 7R.

In addition to their therapeutic applications, an increasing number of studies have focused on developing P2 × 7R-selective positron emission tomography (PET) tracers. PET is a real-time imaging technology that can be used to evaluate biomolecular metabolism, receptor activity, and neurotransmitter activity in vivo. Depending on the selected PET tracer, the activation of P2 × 7R-mediated signaling can be observed in astrocytes and microglia. Purinergic receptors, particularly P2 × 7R, play a causative role in pathological processes in the CNS, especially neuroinflammation associated with neurodegeneration [57, 58].

Several studies have explored the noninvasive imaging of the activation status of microglia and their ability to embody the inflammatory environment based on novel P2 × 7R-selective PET tracers. The microglial membrane-bound neuroinflammation marker [11 C]JNJ54173717 has been used to quantify P2 × 7R expression levels in the human brain in a relatively short time, with sufficient brain uptake [59]. In another study, [11 C]SMW139 was used for PET imaging of neuroinflammation in vivo using an experimental model of autoimmune encephalomyelitis (EAE) [60]. PET radioligands for quantifying P2 × 7R expression levels are potential tools for immunoimaging of the CNS and may provide valuable insights into the monitoring of therapeutic efficacy.

**Table 1:** Commonly used P2 × 7R antagonists/(IC50)

Antagonists	Chemical formula	Weight	Species	Antagonistic activity	Ref
Brilliant Blue G(BBG)	C47H48N3NaO7S2	854.02	Rat IC50 = 10.1 nM Human IC50 = 265 nM	It is a selective antagonist, but restrictedby its poor stability and less desirable pharmacokinetics properties, preventing their use for in vivo studies of the receptoruse for in vivo studies of the receptor	[61]
JNJ-54,175,446	C_18_H_13_ClF_4_N_6_O	440.78	Human IC50 = 8.46 nM Rat IC50 = 8.81 nM Mouse IC50 = 7.8 nM	A potent P2 × 7 antagonist with significant brain penetration and exhibiting dose-dependent P2 × 7 receptor occupancy in the hippocampus of rats and dogs, it has been shown to attenuate dexamphetamine-induced psychomotor hyperactivity and cognitive effects	[62]
JNJ-55,308,942	C17H12F5N7O	425.323	Human IC50 = 10 nM Rat IC50 = 15 nM	JNJ-55,308,942 is orally bioavailable, treatment with JNJ-55,308,942 (30 mg/kg; p.o.) attenuates LPS-induced microglial activation in mice	[54]
A-804,598	C19H17N5	315.372	Human IC50 = 10.9 nM Rat IC50 = 9.9 nM Mouse IC50 = 8.9 nM	In differentiated THP-1 cells that expressed human P2 × 7 receptors, A-804,598 inhibited BzATP stimulated Yo-Pro uptake and release of IL-1β. In 1321N1 cells expressed recombinant rat P2 × 7 receptor, A 804,598 showed high affinity with Kd = 2.4 nM	[10, 56, 63]
SMW139 [^11^ C]SMW139	C_19_H_21_ClF_3_NO_2_ C_18_^11^CH_21_ClF_3_NO_2_	386.824	Mouse IC50 = 24 ± 5.5 nM Human IC50 = 158 ± 44 nM	It has a high affinity for P2 × 7R(Kd = 20.6 ± 1.7 nM), good in vivo stability and the ability to cross the blood-brain barrier, and PET tracers based on it are thought to be useful for detecting the exact role of microglia in CNS disease processes and their activation status	[60, 64, 65]
[^11^ C]JNJ-54,173,717			Human IC50 = 4.2 ± 0.01 nM Rat IC50 = 7.6 ± 0.01 nM		
JNJ-47,965,567	C28H32N4O2	488.6	Human IC50 = 8.3 nM Macaque IC50 = 8.6 nM Dog IC50 = 8.5 nM Rat IC50 = 7.2 nM Mouse IC50 = 7.5 nM	Treatment with the P2 × 7R antagonist JNJ47965567 delayed disease onset, reduced body weight loss and improved motor coordination and phenotypic score in female SOD1G93A mice	[66, 67]

## P2 × 7R: distribution and role in the nervous system

As an ATP-gated nonselective cationic channel, P2 × 7R is widely distributed in the nervous system [17]. P2 × 7R is abundantly expressed in microglia, astrocytes, and neurons in the cerebral cortex (such as the prefrontal cortex and hippocampal areas), spinal cord, other regions of the CNS [12, 68, 69], and the retina [70]. Under normal physiological conditions, low P2 × 7R expression levels do not cause damage; however, under pathological conditions, overexpression of P2 × 7R can cause tissue damage in the nervous system [27, 31, 71], which plays an important role in the development of neurological diseases.

The proliferation of activated spinal microglia is associated with neuropathic pain, and the expression of P2 × 7R is required for microglial activation. After treatment with the selective P2 × 7R antagonist, BBG, the number of activated spinal microglia decreases [18]. During prolonged inflammation or nerve injury, P2 × 7R on the spinal microglia is activated, releasing IL-1β, which regulates downstream cytokines and directly activates injured neurons, ultimately contributing to neuropathic pain [72, 73]. P2 × 7R is upregulated in astrocytes in multiple sclerosis (MS), in which peripheral monocytes invade and damage the CNS [74]. The activation of P2 × 7R causes evident astrocyte activation and neuronal loss by targeting NF-κB p65 in the hippocampal CA1 and CA3 regions. The P2 × 7R antagonists, BBG, A-438,079, and A-740,003, inhibit neuronal death caused by persistent status epilepticus in rats [75]. After traumatic brain injury is activated by P2 × 7R, microglia release microvesicle (MV) like particles, which aggravate nerve cell damage. An antagonist of P2 × 7R (A804598) reduces microglial MV-like particle formation, IL-1β expression levels, p38 phosphorylation, and glial activation in the cerebral cortex, thereby reducing neuronal apoptosis [76]. In a mouse model of AD, microglia were shown to promote reactive oxygen species (ROS) production through P2 × 7R-related stimulation, leading to neuronal damage [77]. Later, it was found that P2 × 7R causes neuronal damage through a combination of ROS production and the downregulation of brain-derived neurotrophic factor [21, 78]. Additionally, in rodent models of AD, the inhibition of P2 × 7R reduces neuronal death. However, significant differential expression of P2 × 7R is not obvious in neurons from older donors with and without AD, and further studies are not possible because of the limited material available in young donor collections [79]. CUMS enhances P2 × 7R-induced microglial activation and neuroinflammation, thereby exacerbating dopaminergic neuronal dysmotility and death in a mouse model of 1-methyl-4-phenyl-1,2,3,6-tetrahydropyridine-induced PD. P2 × 7R inhibition by BBG improves these conditions [13]. Under pathological conditions, such as elevated intraocular pressure in glaucoma, P2 × 7R is upregulated, causing retinal neuronal damage. The P2 × 7R antagonists, BBG and MRS2540, prevent neuronal death induced by age-related macular degeneration, glaucoma, and diabetic retinopathy [80]. In addition to causing tissue damage under pathological conditions, P2 × 7R also plays other roles in the nervous system. P2 × 7R may guide the correct orientation of axons and regulate daily rhythms [81]. P2 × 7R plays an important role in neuronal differentiation and physiology by regulating axonal elongation and synaptic function; however, its role is fundamentally altered under pathological conditions [82].

P2 × 7R plays an important role in the nervous system. P2 × 7R induces neuropathic damage by activating microglia, enhancing the release of inflammatory factors and aggravating neuronal death. The inhibition of P2 × 7R may improve this situation. Therefore, exploring the role of P2 × 7R in neurological diseases is beneficial for the development of treatments targeting P2 × 7R.

## P2 × 7R and neurodegenerative diseases

NDs are progressive neurological disorders characterized by neuronal loss [2, 83]. They mainly include AD, PD, HD, and ALS [6]. These diseases share several common features and molecular mechanisms [84]. A clear explanation of the mechanisms underlying NDs is still lacking; however, neuroinflammation and oxidative stress are crucial for their development [85, 86], and phagocytic defects also play an important role [87]. Neuroinflammation is a local inflammatory response associated with a dysregulated inflammatory response in the nervous system and it plays an important role in many NDs [88]. P2 × 7R preferentially localizes to astrocytes and microglia in the CNS and is activated by high concentrations of ATP released by injured cells in chronic NDs. The activation of P2 × 7R contributes to microglia activation and the proliferation of microglia is involved in multiple inflammatory signaling pathways, such as caspase activation and the assembly of NLRP3 inflammatory bodies. The activation of P2 × 7R-mediated signaling ultimately induces the production of pro-inflammatory cytokines (such as IL-1-β and IL-18) and reactive nitrogen and oxygen species, which cause cellular damage that is superimposed on the original consequences of neurodegeneration [89, 90]. Different stimuli can induce neuroinflammation during the development of various NDs [91, 92]. As markers of the initiation of AD, Aβ and tau oligomers can induce the release of various inflammatory factors from microglia and astrocytes through specific signaling pathways. These processes trigger neuroinflammation, ultimately leading to AD [93, 94]. α-Synuclein in PD and superoxide dismutase 1 (SOD1) in ALS can activate NLRP3 in microglia, causing caspase-1 activation and IL-1β maturation, ultimately leading to the development and progression of related diseases [95, 96]. In the brains of patients with HD, an increased number of reactive microglia and astrocytes, and the release of associated inflammatory factors, lead to neuronal cell death, exacerbating HD progression [97]. Oxidative stress (OS) also plays an important role in the pathogenesis of NDs. An important feature of NDs is that the overproduction of ROS leads to oxidative damage and mitochondrial dysfunction [98, 99], which are common causes of ND progression [100]. In an ALS model, nerve endings were found to be sensitive to ROS, indicating that OS plays an important role in presynaptic decline [101]. OS promotes AD progression by upregulating Aβ formation. In addition, in the early phase of the disease, Aβ formation enhances OS, which further leads to mitochondrial dysfunction and promotes disease progression [102]. The reduced activity of complex I of the respiratory chain in dopamine-containing neurons in the substantia nigra pars compacta of patients with PD may lead to excessive ROS production, causing apoptosis and ultimately aggravating the course of PD [103, 104]. Although neuroinflammation and OS have completely different pathological mechanisms, they interact with each other. In NDs, neuroinflammation is an important cause of ROS overproduction, and the activation of pro-inflammatory pathways promotes OS [105]. ROS also promote the expression and release of pro-inflammatory factors [106]. For example, the activation of microglia and astrocytes in animal and human models of HD produces bound pro-inflammatory cytokines and other toxic substances [107]. These cytokines and toxic substances can further damage peripheral neurons in which OS has already occurred [108–110]. In addition to the above findings, the existence of other links between neuroinflammation and OS requires further investigation. Some phagocytes and microglia in the CNS, and their phagocytic receptors, play important roles in the development of NDs. Phagocytosis of microglia maintains the homeostasis of the nervous system and protects organisms from NDs [111]. For example, TREM2 and CD33 are important innate immune receptors on microglia that regulate the microglial phagocytosis of toxic proteins (e.g., Aβ) and apoptotic cells [112, 113]. Inhibition of TREM2 and activation of CD33 can inhibit microglial phagocytosis, leading to the accumulation of Aβ and myelin debris, which affects the progression of AD and MS [114, 115]. In addition, Toll-like receptors (such as TLR2 and TLR4) are important phagocytic receptors on microglia, and the upregulation of their expression can promote microglial phagocytosis and reduce the accumulation of harmful proteins [116]. In contrast, the lack of Toll-like receptors leads to Aβ accumulation and the exacerbation of AD [117]. Phagocytosis, which is mediated by various receptors, plays an important role in the development and progression of NDs. Further studies on the neuroprotective role of phagocytes may help identify additional targets for the treatment of NDs.

Research has been conducted on the mechanisms of NDs, and some practical treatments have been reported. However, certain molecular mechanisms associated with NDs remain unclear. For example, the exact mechanism of OS in NDs remains unknown, and the key intracellular molecules that coordinate neuroprotective functions in microglia remain poorly understood. Continued exploration of the mechanisms of NDs will benefit the research and treatment of NDs.

Many studies have shown that P2 × 7R is strongly associated with neuroinflammation, and that this association plays an important role in the development and treatment of many diseases. Overall, the upregulated expression of P2 × 7R appears to trigger a range of responses including neuroinflammation, OS, and defective phagocytosis, thereby playing a key role in the progression of NDs. Therefore, P2 × 7R has emerged as a potential therapeutic target for NDs.

### P2 × 7R and AD

At present, excessive Aβ deposition and neurofibrillary tangles (NFTs) are considered the main pathological characteristics of AD, and the main hypotheses for its pathogenesis are the Aβ and tau hypotheses [118, 119].

Several studies have found that neuroinflammation associated with AD induces altered distribution patterns of P2 × 7R, which is upregulated in microglia near Aβ plaques during the advanced and late stages of AD when there is significant microglial proliferation, and that P2 × 7R activation promotes the migration of microglia to senile plaques, but reduces their phagocytic capacity [120]. In addition, Aβ causes an increase in Ca^2+^ concentration in microglia via P2 × 7R and, consequently, triggers ATP release from these cells, activating NLRP3 inflammatory vesicles and leading to increased IL-1β release, which is involved in the development of AD via multiple pathways. This effect may be accomplished through the inhibition of the action of α7 nicotinic acetylcholine receptors by Aβ [121]. Many studies have shown that IL-1β promotes the formation of Aβ plaques, causes the deposition of hyperphosphorylated tau proteins, impairs synaptic plasticity, and ultimately leads to neuronal death [122]. Some studies have also offered alternative views on the role of IL-1β in the development of AD, with acute stimulation of IL-1β leading to neuroprotection and subacute stimulation of IL-1β leading to neurotoxicity [123]. Additional studies have shown that Aβ is dependent on the P2 × 7R and induces mitochondrial toxicity in microglia through various mechanisms that affect mitochondrial autophagy, causing decreased ATP production and increased OS, ultimately leading to impaired microglial phagocytosis and promoting neuroinflammation and AD development [124]. Another study that analyzed the mechanism of action of P2 × 7 deletion in tau mice further demonstrated that, while P2 × 7-deficiency had a moderate effect on tau phosphorylation, it improved the neuroinflammatory response by reducing microglial activation and the production of related inflammatory markers. Importantly, P2 × 7 deletion in tau mice improved long-term synaptic plasticity in the hippocampus. These results indicate that P2 × 7 deficiency has major beneficial effects in both amyloid and tau contexts [125].

In addition to its localization in the microglia, P2 × 7R is expressed at low levels in astrocytes and oligodendrocytes. Astrocytes and oligodendrocytes mediate necrosis and apoptosis through the release of pro-inflammatory cytokines and chemokines, ROS, and excitatory neurotoxic transmitters, glutamate and ATP, which affect neuronal cell development and mediate cellular damage during AD and other chronic neurodegenerative processes [126].

Overall, the mechanism of action of P2 × 7R in AD still needs to be thoroughly investigated, and exploring the interrelationships between P2 × 7R and inflammatory responses, neuronal damage, and Aβ deposition may provide more insights for the development of P2 × 7R-based therapeutic strategies for AD. Furthermore, additional preclinical studies are warranted to confirm the efficacy and safety of P2 × 7R as a potential target for AD treatment, and these experiments, including animal models and cellular experiments, will allow us to systematically evaluate the effects of P2 × 7 antagonists on reducing inflammation, neuroprotection, and cognitive function improvement.

### P2 × 7R and PD

PD is characterized by the degeneration of nigrostriatal dopaminergic (DA) neurons, reduced dopamine levels in the striatum, and the formation of abnormal protein aggregates in neurons called Lewy bodies, the main component of which is α-synuclein [127]. The involvement of P2 × 7R and neuroinflammation in the pathogenesis of PD has been supported by extensive studies [128, 129]. In *de novo* PD patients, increased mRNA and protein expression levels of the P2 × 7R/NLRP3 inflammasome components have been observed in peripheral blood mononuclear cells [46]. This finding is consistent with the hypothesis that excessive microglial activation and chronic neuroinflammation lead to nigrostriatal and striatal DA neuron degeneration in patients with PD.

P2 × 7R may activate microglia via the p38 MAPK pathway, leading to nigrostriatal DA neuronal deficiency. Studies have indicated that when LPS-injected rats are treated with BBG, p38 MAPK activation is reversed, microglial activation is attenuated, and the loss of DA neurons is reduced in the substantia nigra [130]. Another study demonstrated that the P2 × 7R/PI3K/AKT signaling pathway is potentially involved in the release of cathepsin L from aberrantly activated microglia and this ultimately affects the survival of neurons in PD [131]. Activation of NADPH oxidase (PHOX) leads to the production of superoxide and OS, is associated with the release of the excitotoxic neurotransmitter glutamate [35], and is thought to be partially responsible for the death of DA neurons in patients with PD. Studies have shown that α-synuclein is associated with PHOX activation, and its pathway of action may be that α-synuclein activates the PI3K/AKT pathway by stimulating microglial P2 × 7R, which ultimately causes OS [132]. 2,4,5-Trihydroxyphenethylamine (6-OHDA) can be used to establish an animal model of PD [133], and treatment with the P2 × 7R antagonists, A-438,079 or BBG, significantly prevents the 6-OHDA-induced depletion of striatal dopamine stores and reverses behavioral deficits [134, 135]. However, not all experimental results support the view that P2 × 7R activity or its inhibition promote the survival of DA neurons, in both in vivo and in vitro models of PD. Other experimental results from different PD models indicate that P2 × 7R plays a dual role in different cellular response cascades, leading to neuronal death. For example, in a PD model induced by rotenone, P2 × 7 channel current density increases after treatment, and the activation of P2 × 7R in astrocytes inhibits TNF-α secretion [136].

In general, most previous studies have explored the immunological role of P2 × 7R, as it is thought to be closely associated with microglia and astrocytes and plays an important role in the regulation of neuroinflammatory responses; however, the pathogenesis of PD involves multiple factors, such as OS, neuroinflammation, and abnormal mitochondrial function. Future studies should explore the interactions between P2 × 7R and these factors to improve our understanding of the mechanisms underlying PD development.

### P2 × 7R and HD

HD is a fatal ND inherited in an autosomal dominant manner and characterized by motor, cognitive, and psychiatric dysfunctions [137]. HD is caused by amplification of a CAG repeat in exon-1 of the Huntington gene (*HTT*), which is translated into a polyglutamine (polyQ) extension in the HTT protein, ultimately leading to neuronal dysfunction and loss through protein interactions that affect gene transcription, cellular endocytosis, and metabolism. The pathology of HD is characterized by brain atrophy and neuronal death occurring mainly in the cortex and striatum [138].

By analyzing the level and function of the P2 × 7 purinergic receptor in mouse and HD cell models, researchers found that the level of P2 × 7R were elevated in the soma and terminals of neurons expressing mutant HTT and further mediated alterations in calcium homeostasis. In vivo administration of BBG prevents apoptosis in untreated HD mice and in HD neuronal cultures lacking glial cells, strongly suggesting the potential of P2 × 7R antagonists in the treatment of HD [139]. However, with further studies on the localization of P2 × 7R in the nervous system in recent years, more evidence is required to confirm the expression and function of P2 × 7 in neurons. In a recent study, researchers evaluated the expression and function of P2 × 7R in two genetic models of HD (ST14A/Q120 rat striatal cells and R6/2 mice) using the P2 × 7R agonist BzATP. Experimental results have shown that P2 × 7R expression levels and activity are altered in the presence of HD mutations and that the effect of BzATP observed in corticostriatal slices strongly depends on presynaptic A1R activation, providing a unique perspective on the mechanism of action of P2 × 7R in HD [140]. In a study of the status of P2 × 7R in the brains of patients with HD, increased levels of total *P2 × 7R* transcription and changes in its splicing were observed. Protein levels of the full-length form of P2 × 7R (P2 × 7R-A) and the naturally occurring human variant lacking the C-terminal region, P2 × 7R-B, were both upregulated [141].

In general, we can assume that, during the development of NDs, signaling crosstalk occurs between neurons, glial cells, and microglia, with P2 × 7R acting as a central hub for neuroinflammation and participating in a series of signaling pathways. This ultimately causes the release of pro-inflammatory signaling factors, leading to chronic neuroinflammation and damaging effects of the excitotoxic neurotransmitter, glutamate, and OS, resulting in the progressive degeneration of nerve cells. Therefore, the nature of P2 × 7R alterations in HD warrants further exploration.

It is important to note that HD is a complex disorder for which the etiology and pathophysiology remain incompletely understood. Many changes related to purine metabolism occur in the CNS, skeletal muscle, and heart of HD patients and in animal and cellular models; however, changes in these systems may involve different mechanisms. An in-depth understanding of the relationship between P2 × 7R and the course and symptoms of HD, and the changes in P2 × 7R in patients with HD, in terms of the different subtypes, mutations, and levels of expression, are important for treating HD and slowing disease progression.

### P2 × 7R and ALS

ALS is a fatal ND caused by the loss of motor neurons (MNs) in the cerebral cortex, brainstem, and spinal cord [142]. The disease causes progressive weakness and atrophy of the muscles of the limbs, trunk, chest, and abdomen, which affects movement, communication, swallowing, and breathing, ultimately leading to death [143].

Although the pathophysiological mechanisms of ALS are not fully understood, the influence of genetic factors has been widely recognized, and mutations in genes such as *SOD1*, *C9ORF72*, and *FUS* have been associated with the development of ALS [142]. Several mouse models of ALS-associated mutations have been developed. Previous studies have shown that the pathogenesis of ALS includes calcium-dependent excitotoxicity, altered mitochondrial ultrastructure, and ROS overproduction [144], which are associated with the upregulation of P2 × 7R in microglia and macrophages in affected regions [145].

Purinergic ionotropic P2 × 7Rs play a dual role in the progression of NDs by acting at different cellular and molecular levels, as confirmed in ALS studies. Many studies have suggested that P2 × 7R plays a partial role in ALS progression [146], because the application of the P2 × 7R antagonist BBG was found to reduce body weight loss in *SOD1*^G93A^ mice of both sexes [50]. This negative effect may be due to the increased extracellular ATP levels around the lesion. Microglia and astrocytes are the two major contributors to motor neuron dysfunction in ALS [56]. The activation of P2 × 7R in microglia, which is involved in the assembly of NLRP3 inflammatory vesicles and the activation of caspases, ultimately causes increased IL-1β release, which induces COX-2 expression and the production of downstream pathogenic mediators [145]. In addition to directing the conversion of microglia to a pro-inflammatory phenotype, P2 × 7R is thought to be a major receptor for inducing the neurotoxic phenotype of astrocytes upon endogenous ATP stimulation, contributing to their toxicity toward MNs. Repeated stimulation of P2 × 7R with the P2 × 7-selective agonist BzATP triggers the neurotoxic phenotype of astrocytes, which was further confirmed by the inhibition of ATP and BzATP by BBG [147].

In addition to mediating the conversion of microglia and astrocytes into a neurotoxic phenotype, P2 × 7R located on NSC-34 MNs is believed to mediate the ATP-induced rapid release of aggregated *SOD1*^G93A^ from NSC-34 cells, which is blocked by AZ10606120, a high-affinity, potent, and selective P2 × 7R antagonist [148].

Although the above results suggest that P2 × 7R has a negative impact on ALS, findings based on P2 × 7R knockout in heterozygous and homozygous *SOD1*^G93A^ mice provide new evidence that structural deletion of P2 × 7R exacerbates disease progression in ALS [149]. Another study indicated that the stimulation of P2 × 7R with BzATP improved the innervation and metabolism of myofibers and induced the proliferation and differentiation of satellite cells, thereby preventing denervation and skeletal atrophy in *SOD1*^G93A^ mice [150]. These studies have revealed the complex dual role of P2 × 7R in ALS and the highlight the multifactorial and multisystemic nature of this disease.

P2 × 7R provides neuroprotection in early asymptomatic ALS, while promoting neuronal degeneration in the pre-symptomatic and symptomatic stages. Thus, the use of various P2 × 7R antagonists may be a potential strategy for the treatment of ALS. These partially contradictory findings also suggest that we need to carefully consider the time of administration, sex, and disease progression in future P2 × 7R antagonist trials for ALS. We also need to develop and apply a wider variety of animal models of ALS with more specific P2 × 7R antagonists, and deepen our understanding of the exact role of P2 × 7R in the pathological processes ALS to identify potential therapeutic targets. ALS is a complex disease, and future studies should explore multimodal therapeutic strategies, including combinations of P2 × 7R antagonists with other drugs or therapies. These strategies may target multiple pathological pathways for simultaneous intervention to provide more effective therapeutic outcomes.

### P2 × 7R and MS

MS is a chronic autoimmune disease of uncertain etiology. It is characterized by focal lesions with neuroinflammation, CNS demyelination, oligodendrocyte death, and axonal damage. It occurs in young people aged 20–50 years, and is two to three times more common in females than in males [151]. The EAE model is a common animal model of MS.

P2 × 7R has attracted the attention of MS researchers because of its higher expression levels in the microglia of postmortem spinal cord specimens from patients with MS than in their non-MS counterparts [145]. Researchers further investigated the time course of microglial activation in the brains of rats with EAE, and the results showed that microglial activation occurs in the brains of immunized rats at a very early stage of EAE, long before neurological symptoms develop [152].

In addition to microglia, astrocytes are activated during the early stages of EAE and exhibit increased P2 × 7R expression levels. In a study of P2 × 7R protein expression in the brains of EAE rats, Tomasz et al. found that increased levels of the astroglial marker, glial fibrillary acidic protein, in brain homogenates and the astroglial fraction were consistent with astrocytic pool of cells [153]. Administration of the P2 × 7R antagonist, BBG, significantly decreases astrogliosis, which may be involved in the pathogenesis of MS/EAE at a very early stage through purinergic/glutamatergic mechanisms [154].

Genetic studies have provided additional evidence that P2 × 7R mediates neuroinflammation and is involved in the pathogenesis of MS [155]. A rare *P2 × 7* genetic variant, Arg307Gln, has been found to have a protective effect against MS, with heterozygotes showing nearly absent pro-inflammatory ‘pore’ function. This variant is believed to decrease the secretion of pro-inflammatory cytokines from activated microglia/macrophages and lessen the inflammation associated with acute relapse and progressive disease [156].

Previous research on the treatment of MS has focused on neuronal activity and immune cell responses; however, a growing number of studies have focused on the factors influencing P2 × 7R expression and function, as well as genetic variation and its association with MS risk. Such studies may increase our understanding of the differences in P2 × 7R function between individuals and their relevance to the development of MS. Two single- nucleotide polymorphisms in the *P2 × 7R* gene have been found to correlate with disease severity in MS [157]. Further exploration of the genetic background of patients with MS may allow P2 × 7R to be used as a target for designing individualized treatment and rehabilitation intervention programs for patients with MS.

**Fig. 4:**
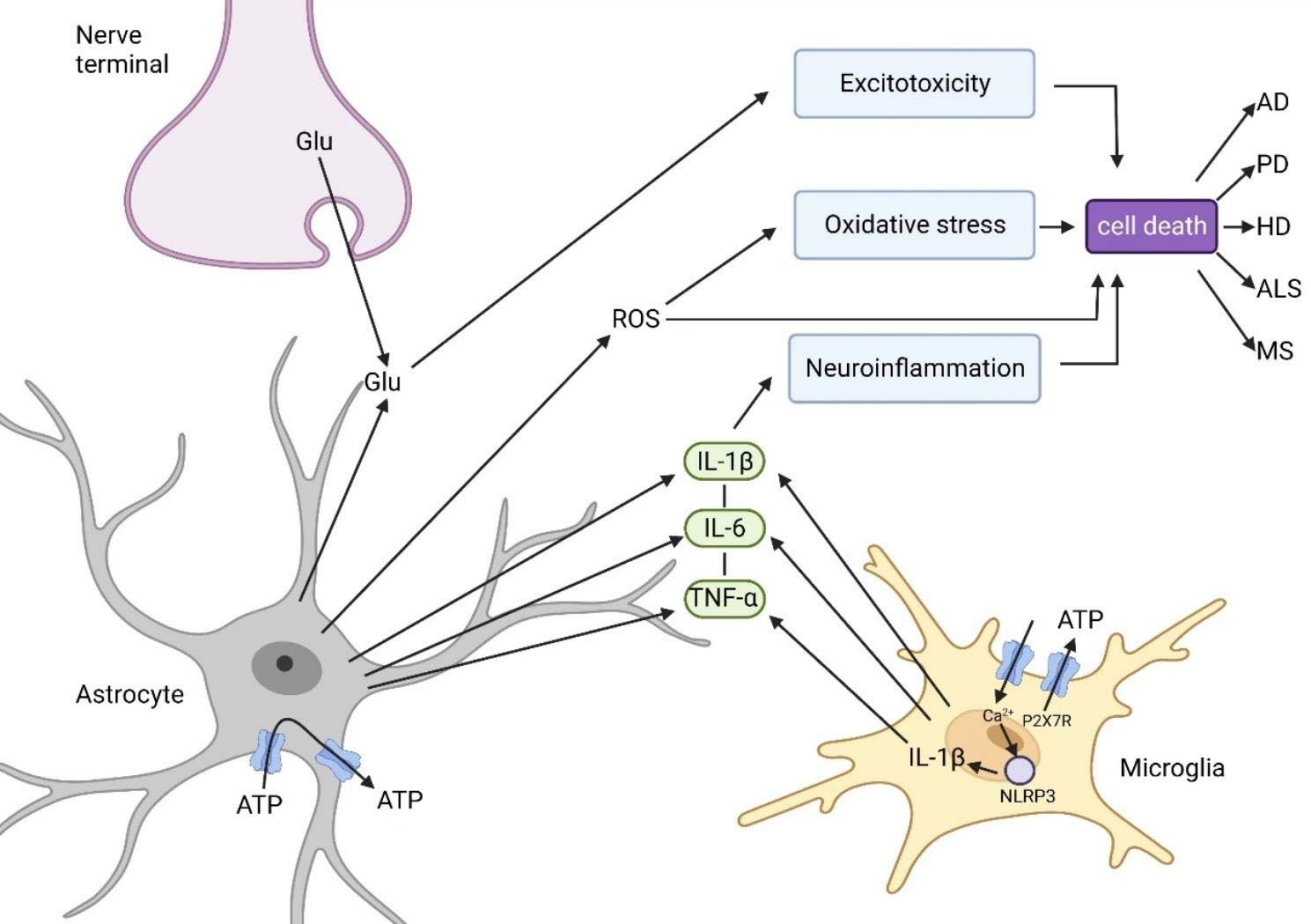
Microglia, astrocytes, and nerve terminals are involved in pathways associated with neurodegenerative diseases. Activation of P2 × 7R causes an increase in Ca^2+^ concentration in microglia, which activates NLRP3 and leads to the release of IL-1β. P2 × 7R is expressed on microglia and also expressed at a low density on astrocytes, mediating the release of ATP. Glu released from the nerve terminal and astrocytes causes cell death via excitotoxicity. Reactive oxygen species (ROS) released by astrocytes cause cell death directly or indirectly through oxidative stress. Microglia and astrocytes both release IL-1β, IL-6, and TNF-α, and these cytokines cause cell death through neuroinflammation. These above-mentioned processes are ultimately involved in the development and progression of neurodegenerative diseases. Figure 4 has been modified based on Sperlagh B et al. [32]

Overall, the level of P2 × 7R expression is closely associated with the onset and progression of NDs (Fig. 4). Reducing P2 × 7R levels or blocking P2 × 7R activation may inhibit the mechanisms and pathways associated with NDs, thereby achieving the goal of treating these diseases.

## Conclusions

The role of P2 × 7R in the occurrence and development of NDs has become clearer owing to extensive research on this topic. P2 × 7R, a subtype of the P2X purinergic receptor family, is activated by ATP and its derivatives. The activation of P2 × 7R leads to the release of pro-inflammatory factors from microglia and astrocytes through various signal transduction pathways, leading to the development of neuroinflammation. Neuroinflammation is one of the primary mechanisms underlying the development of NDs. Moreover, in the absence of ATP, P2 × 7R functions as a scavenger receptor in the phagocytosis of neuroprotective microglia. Phagocytosis of toxic proteins and apoptotic cells by phagocytes in the CNS helps reduce the effects of NDs on the body. P2 × 7R affects multiple NDs by regulating protective autophagy mechanisms.

Thus, P2 × 7R is an important target for the treatment of NDs. However, many aspects of its involvement in NDs remain unknown. For example, it remains unclear how P2 × 7R plays a role in the neuroprotective function of astrocytes. Furthermore, in the absence of ATP, the signaling molecules and related signaling pathways by which P2 × 7R affects phagocytosis by phagocytic cells and microglia in the nervous system influence the occurrence and development of NDs.

There is a need to discover other related signaling molecules and pathways based on the findings of previous studies. The exploration of signaling molecules and pathways related to P2 × 7R may inspire us to consider the function and therapeutic significance of P2 × 7R antagonists. We expect that additional P2 × 7R-related signaling pathways and P2 × 7R-targeted antagonists will be discovered in the future, thereby facilitating the treatment of NDs.

## Data Availability

The data supporting the conclusion of this review have been included within the article.

## Abbreviations

α-Syn: α-synuclein
6-OHDA: 2,4,5-trihydroxyphenethylamine
AD: Alzheimer’s disease
ALS: Amyotrophic lateral sclerosis
ATP: Adenosine 5′- triphosphate
BBB: Blood-brain barrier
BzATP: 2′,3′-O-(4-benzoylbenzoyl)-adenosine 5′-triphosphate
CNS: Central nervous system
CTSL: Cathepsin L
CUMS: Chronic unpredictable mild stress. Aβ,amyloid protein
DA: Nigrostriatal dopaminergic
EAE: Experimental autoimmune encephalomyelitis
ERK: Extracellular signal-regulated Kinase
HD: Huntington’s disease
HTT: Huntington gene
JNK: C-Jun N-terminal kinase
MAPKs: Mitogen-activated protein kinase
MN: Motor neurons
MS: Ultiple sclerosis
MV: Microvesicle
nAChRs: Nicotinic acetylcholine receptors
NDs: Neurodegenerative diseases
NFT: Neurofibrillary tangles
oATP: Oxidized ATP
OS: Oxidative stress
PD: Parkinson’s disease
PET: Positron emission tomography
ROS: Reactive oxygen species
SOD1: Superoxide dismutase
TM: Transmembrane
TNF-α: Tumor necrosis factor-α
